# Morphogenesis and Production of Enzymes by *Penicillium echinulatum* in Response to Different Carbon Sources

**DOI:** 10.1155/2014/254863

**Published:** 2014-04-30

**Authors:** Willian Daniel Hahn Schneider, Laísa dos Reis, Marli Camassola, Aldo José Pinheiro Dillon

**Affiliations:** Enzyme and Biomass Laboratory, Institute of Biotechnology, University of Caxias do Sul, 1130, Francisco Getúlio Vargas Street, 95070-560 Caxias do Sul, RS, Brazil

## Abstract

The effect of different carbon sources on morphology and cellulase and xylanase production of *Penicillium echinulatum* was evaluated in this work. Among the six carbon sources studied, cellulose and sugar cane bagasse were the most suitable for the production of filter paper activity, endoglucanases, xylanases, and **β**-glucosidases. However, sucrose and glucose showed **β**-glucosidase activities similar to those obtained with the insoluble sources. The polyacrylamide gels proved the enzymatic activity, since different standards bands were detected in the media mentioned above. Regarding morphology, it was observed that the mycelium in a dispersed form provided the greatest enzymatic activity, possibly due to greater interaction between the substrate and hyphae. These data are important in understanding the physiology of fungi and could contribute to obtaining enzyme with potential application in the technology of second generation ethanol.

## 1. Introduction

Currently, there is great interest in cellulases and xylanases, due to their broad applications in food and feed, the textile industry, and the pulp and paper industries. However, the greatest potential application of these enzymes is the hydrolysis of cellulose and xylan, available in large quantities in lignocellulosic residues from agricultural, urban, and industrial solid waste. Syrups of glucose and xylose obtained from the hydrolysis of lignocellulosic biomass could be used for various biotechnological purposes, especially the production of ethanol [1, 2].

Cellulases and xylanases are enzymatic complexes produced and excreted primarily by fungi and bacteria. A cellulolytic enzyme system is a complex of enzymes composed of endoglucanase (endo-1,4-*β*-D-glucanase, EC 3.2.1.4), exoglucanase (1,4-*β*-D-glucan-cellobiohydrolase, EC 3.2.1.91), and *β*-glucosidase (*β*-D-glucoside glucanohydrolase, cellobiase, EC 3.2.1.21) that acts synergistically to degrade cellulosic substrate [3–5].

Biodegradation of xylan by xylanases requires the action of several enzymes. They are endoxylanases (EC 3.2.1.8) which randomly hydrolyze xylan backbone, yielding a mixture of xylooligosaccharides; xylosidases (EC 3.2.1.37), which liberate xylose oligosaccharides short; *α*-L-arabinofuranosidases (EC 3.2.1.55) which remove L-arabinofuranose side chain; *α*-D-glucuronidases (EC 3.2.1.139), which hydrolyze the residue methyl D-glucuronate; acetyl xylan esterases (EC 3.1.1.72), which hydrolyze acetate groups of the main chain; feruloyl/coumaryl esterases (EC 3.1.1.73) which hydrolyse the corresponding aromatic acids residues attached to arabinofuranoside [6].

The ability to replace gasoline in internal combustion engines has pointed ethanol as a viable solution to meet current energy demands and reduce the emission of greenhouse gases. Currently, the main crops used for ethanol production are sugar cane and corn. However, they have the disadvantage of being used for human food and livestock, in addition to requiring fertile land and pesticide use for cultive [7].

In this context, lignocellulosic biomass has enormous potential to contribute to this technology due to the wide availability and low cost of this raw material. The production of ethanol from lignocellulosic residues can significantly increase fuel production without increasing the cultivated area. However, the high cost of production of the enzyme complex and the need for pretreatment of lignocellulosic material is a limit the development 6 of a technology for ethanol production on an industrial scale [8].

The ability of filamentous fungi secreting a pool of proteins has motivated their intensive use for the production of industrial enzymes. The* P. echinulatum* is also among the organisms with high potential for production of cellulases [9]. Mutants are capable of secreting cellulase activity of filter paper greater than 2 IU/mL, employing 1% cellulose as carbon source, with values greater than 30 U/dry weight in solid state fermentation using sugarcane bagasse [3]. The importance of the enzymatic complex of* P. echinulatum* is also due to a balanced ratio of FPA and *β*-glucosidase, a fact relevant to the hydrolysis of cellulose [10]. However, the morphology and the mechanisms involved in cell growth and product formation are little known for* P. echinulatum*. Factors that influence the production of enzymes are the morphology of the organism during growth. In submerged cultures, the morphology of filamentous microorganisms typically varies between pellets and dispersed forms, depending on their growing conditions and genotype of the strain of the organism [11].

Therefore, it is essential to carry out studies of relationships between morphology, growth, and enzyme production to meet the physiology of the fungus and thus plan efforts to improve processes enable production of these enzymes. In this context, in the present study, we evaluated the effect of six different carbon sources on morphogenesis and enzyme production by* P. echinulatum* S1M29.

## 2. Materials and Methods

### 2.1. Strain

For the production of cellulases and xylanases was used S1M29 mutant strain of* Penicillium echinulatum* obtained from 9A02S1 strain (microorganism deposited at German collection of microorganisms and Cell Cultures-DSM 18942). The S1M29 strain was obtained by employing hydrogen peroxide mutagenesis and selection of mutants in medium supplemented with 2-deoxyglucose [12]. The strain 9A02S1 was obtained after several steps of mutagenesis, characterized by being a mutant partially derepressed of glucose [9].

### 2.2. Growth and Maintenance of Strain

The strain was grown and maintained in 100 mL of cellulose agar (agar-C) consisting of 40 mL of swollen cellulose, 10 mL of mineral solution containing (in g·L^−1^) KH_2_PO_4_, 20; (NH_4_)_2_SO_4_, 13; CO(NH_2_)_2_, 3; MgSO_4_·7H_2_O, 3; CaCl_2_, 3; FeSO_4_·7H_2_O, 0.050; MnSO_4_·H_2_O, 0.0156; ZnSO_4_·7H_2_O, 0.014; and CoCl_2_. 0.0020., 0.1 g proteose peptone (Oxoid L85), 2 g agar, and 50 mL of distilled water. The strain was grown in tubes inclined with C-agar for 7 days at 28°C until the formation of conidia and then stored at 4°C [9].

### 2.3. Production of Cellulases and Xylanases

The culture medium for the production of cellulases and xylanases consisted of 0.2% (w/v) peptone, 0.05% (w/v) Prodex, 1% (w/v) of the power carbon, 0.1% (w/v) Tween 80, 0.002% (v/v) antibiotic ciprofloxacin (Proflox - EMS/S), and 5% (v/v) solution of mineral [13] and distilled water to complete a final volume of 100 mL.

With the objective of evaluating the effect of different carbon sources on morphology and enzymatic activities of the mutant S1M29 of* P. echinulatum*, were used as carbon source commercial sucrose, glucose, glycerol, cellulose* Celuflok *E, untreated elephant grass, and sugar cane bagasse pretreated by steam explosion.

The elephant grass was dehydrated at 60°C for three days and then crushed with the forage chopper (Trapp 70), yielding a grain size between 200 and 4 mesh. The elephant grass used in the experiment is from Nova Petropólis city, Rio Grande do Sul, Brazil. Samples of sugar cane bagasse pretreated by steam explosion were obtained from Vale do Rosario Farm, Morro Agudo/SP. This plant uses sudden decompression with full discharge of the reactor. The operation is performed in cycles with a load of 840 kg sugar cane bagasse and 50% moisture without prior treatment. The operation utilizes saturated steam (25 kg/cm^2^) with automatic injection (1 min ramp), so that the reactor operates at 16 kg/cm^2^ for about 7 min. After this period, the reactor is discharged through a gate valve and blown sugar cane bagasse is sent to a silicone where the expansion occurs and collection. This pretreated sugar cane bagasse is commonly prepared and marketed as a component of animal feed.

Erlenmeyer flasks 500 mL containing 100 mL of the production medium were inoculated with a suspension of 1 × 10^5^ conidia per mL and kept at 28°C reciprocal shaking at 180 rpm for 120 hours. The experiment was performed in triplicate and samples were collected at intervals of 24 hours. The samples were kept under refrigeration with sodium azide 20% (w/v).

### 2.4. Determination of Mycelial Growth of Dry Biomass

The mycelial growth was determined by dry weight of the fungal biomass. For this, 60 mL of culture was centrifuged for 9.600 g for 20 minutes, after removal of the supernatant, the pellets were washed three times with distilled water (10 mL), and drying at 40°C under reduced pressure until constant weight. For the experiments with insoluble 16 carbon sources, it was calculated the difference between the total dry weight of solids (including mycelium and carbon source) and the dry weight of residual carbon source (cellulose). The residual cellulose content in the samples was determined by the method of Updegraff (1969) [14] modified by Ahamed and Vermette (2008) [15]. This method uses 10 mL of cultive medium, which is centrifuged at 10,000 g for 20 minutes. After the supernatant was removed. The resulting pellets were suspended in 3 mL of a solution of acetic acid and nitric acid, leaving the mixture in a bath at 100°C for 30 minutes. After cooling and centrifugation at 3000 g for 20 minutes, the pellets are washed with distilled water (10 + 10 mL) in a dry filter paper and weighed. The residual pulp is dried at 50°C for 48 hours.

### 2.5. Morphological Analysis

Different morphological analyses were performed on mycelium samples obtained during the cultives using different carbon sources to assess the morphology of the strain of* P. echinulatum* S1M29. The morphology of the mycelium classified according to Cox et al. (1998) [11] may be a dispersed mycelium, differentiating between freely dispersed and aggregated (or mycelial clumps) or pellets.

#### 2.5.1. Optical Microscopy

The study of morphogenesis in optical microscopy was carried out with suspensions of mycelia grown on different carbon sources in 0, 24, 48, 72, 96, and 120 hours of culture. Volumes of 50 *μ*L of suspensions of mycelia were stained with 50 *μ*L of solution of 1% congo red, observed under an optical microscope Bell Photonics model and photographed with a camera microscopic Dino-Eye digital, AM423X model. This step was assessed primarily using general morphology of the mycelium.

#### 2.5.2. Scanning Electron Microscopy

The analysis of scanning electron microscopy was performed according to the methodology used in the Materials Characterization Laboratory (LCMAT) of the University of Caxias do Sul. The methodology is based on a process called magnetron-sputtering plasma, known as (PVD) physical vapour deposition.

Aliquots of each sample of the mycelium of different culture media were deposited on aluminum stubs and were dehydrated at 60°C for withdrawing maximum moisture of the samples.

Then the samples were kept in the evacuated until the pressure base of 5 × 10^−12^ mbar. During pressure decrease, the gas chamber and the surface were removed, and the low-pressure volatile components and remaining water samples were also taken.

After injected with a thin atmosphere of argon, reaching pressure of 1 × 10^−1 ^mbar. The partial pressure of argon is the difference between the working pressure and the base pressure.

The source voltage and current were ligated with 20 mA current and voltage of 350 V. The ionized argon up and start the process of PVD, which lasted 2.5 minutes. Atoms pulling a gold target are accelerated toward the sample surface, generating nanoscale layers and uniform and conductive metallic gold.

Prepared samples were observed and photographed in a scanning electron microscope, model Supercan Shimadzu SSX-550.

With scanning electron microscopy sought primarily to evaluate the morphology of branching hyphae as well as its ends. In this step, the mycelia were analyzed for 48 and 72 hours of culture.

### 2.6. Determination of Enzyme Activity

The total cellulase activity was measured using the filter paper activity (FPA), adapted from Ghose (1987) [16] according to Camassola and Dillon (2012) [17]. The endoglucanases activities were carried out according to Ghose (1987) [16] with modifications. *β*-glucosidase activity was carried out using a methodology adapted from Daroit et al. (2008) [18], using *ρ*-nitrophenyl-*β*-D-glucopyranoside as substrate. Xylanases activities were performed according to Bailey et al. (1992) [19], using oat spelled xylan (1% w/v). After five minutes of incubation, the reaction was stopped by adding 300 *μ*L DNS.

FPA units, xylanases, and endoglucanases were taken as the amount of enzyme capable of releasing 1 *μ*M of reducing sugar per minute. One unit of *β*-glucosidases (*ρ*-nitrophenyl-*β*-D-glucopyranoside) was taken as the amount of enzyme capable of releasing 1 *μ*M of *ρ*-nitrophenyl per minute.

### 2.7. SDS-PAGE of Total Proteins

To determine the molecular weight of cellulases, was performed polyacrylamide gel electrophoresis containing 0.1% sodium dodecyl sulfate (SDS-PAGE). The separating gel was prepared with 12% (w/v) while the stacker gel was prepared 4% (w/v), using methods described by Laemmli (1970) [20]. The samples, after preparation with sample buffer containing 5% (w/v) *β*-mercaptoethanol, were applied to the gel for the electrophoretic tank in vertical Bio Rad Protean II xi Cell 2-D at 200 V. Were applied 250 *μ*L of sample from enzymatic cellulase broth in the gel, also as sugar cane bagasse pretreated by steam explosion and untreated elephant grass and 1000 *μ*L of glucose enzyme broths, sucrose and glycerol.

Revelation of the bands from the gel was performed by incubating the gel for 30 minutes in a solution of 0.2% Coomassie Brilhant Blue G 250, 50% ethanol, and 10% acetic acid. After the gel was washed with distilled water and immersed in a solution of 50% ethanol and 10% acetic acid for 30 minutes, the entire development process was carried out under agitation of 50 rpm, until bands were visualized.

### 2.8. Analysis of Results

Graphs and data analysis were done in the software PrismGraphPad. The results were analyzed by ANOVA and Tukey posttest at *P* < 0.05.

## 3. Results and Discussion

### 3.1. Changes in pH and Growth

**Figure 1:**
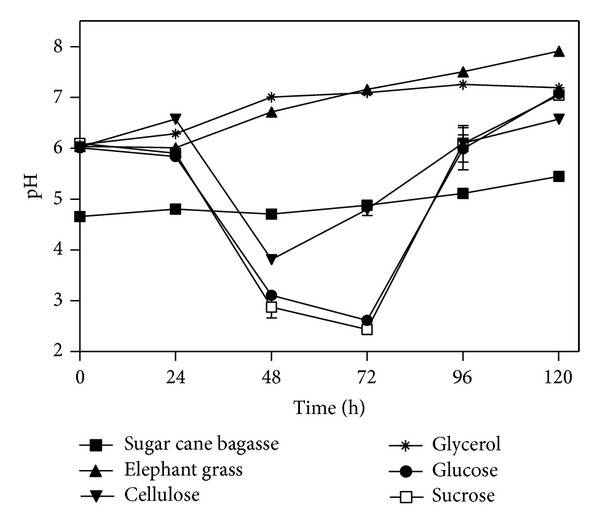
pH variation versus time of* P. echinulatum* S1M29 in submerged cultive, using different carbon sources.

The experiment using six different carbon sources showed variations in pH during the 120 hours of culture for each carbon source used.  Figure 1 shows that the pH of the culture medium using sugar cane bagasse pretreated by steam explosion remained virtually unchanged during the whole cultive. It is suggested that some component of sugar cane bagasse, such as salts, has a buffering effect of the medium. The culture media using cellulose, glucose, or sucrose demonstrated a decrease in pH after 24 hours of culture, suggesting that, with these carbon sources, there is an increased metabolism of the fungus. The pH increases again after 48 hours of cellulose and sucrose cultive and after 72 hours of glucose cultive.

In filamentous fungi cultures with no pH control, there is a decrease in pH during growth on substrates containing carbohydrates and, after exhaustion of the carbon source, a rise in pH is observed (Bailey and Tähtiharju, 2003) [21]. Sternberg and Dorval (1979) [22] suggest that a decrease in pH occurs due to consumption of ammonia present in the production medium in the form of ammonium sulfate, with release of H^+^ in the medium.

The rapid metabolism in the medium employing cellulose as carbon source may also have occurred parallel to faster increase in pH. These data are consistent with studies of Sternberg and Dorval (1979) [22], since they also found that pH can be a parameter indicative of the intensity of metabolism.

The pH of the culture medium using elephant grass untreated increased gradually from the beginning to the end of cultive. Similar behavior was observed for the culture medium with glycerol up to 96 hours of culture, when the pH is replaced by a small drop.

**Figure 2:**
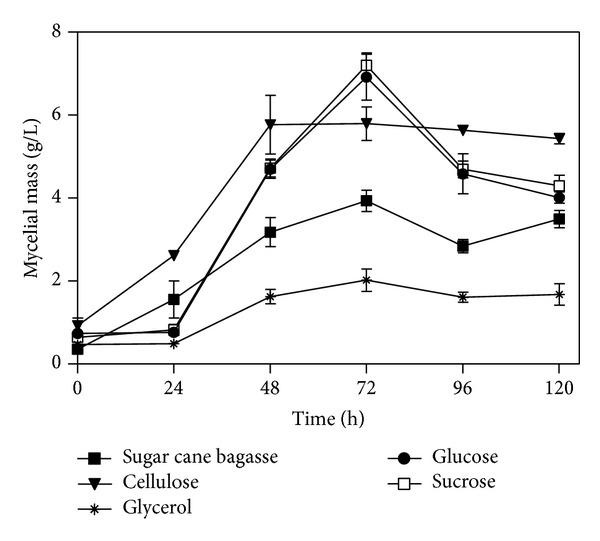
Mycelial mass versus time of* P. echinulatum* S1M29 in submerged cultive, using different carbon sources. Due to the difficulty of measuring the growth on cultive formulated with insoluble substrates, it was not quantitate the growth in the cultive formulated with elephant grass.

The pH profiles showed respect to growth (Figure 2), which allows us to suggest that the increase in cellular metabolism to the production of biomass gives a decrease in pH, as reported by Sternberg and Dorval (1979) [22] for* Trichoderma reesei*. Besides the “take-up” of ammonia, the decrease in pH may have been due to production of organic acids, as suggested by Blandino et al. (2002) [23] and Botella et al. (2009) [24] during production of enzymes by* Aspergillus awamori.* According to these authors, when the concentration of reducing sugars decreases, the pH increases, probably due to assimilation of organic acids. Having the means which were employed in the elephant grass or glycerol as carbon sources resulted in a growth of little significance, with increasing pH.

### 3.2. Enzymatic Activities

#### 3.2.1. Activities of FPA (Filter Paper Activity)

**Figure 3:**
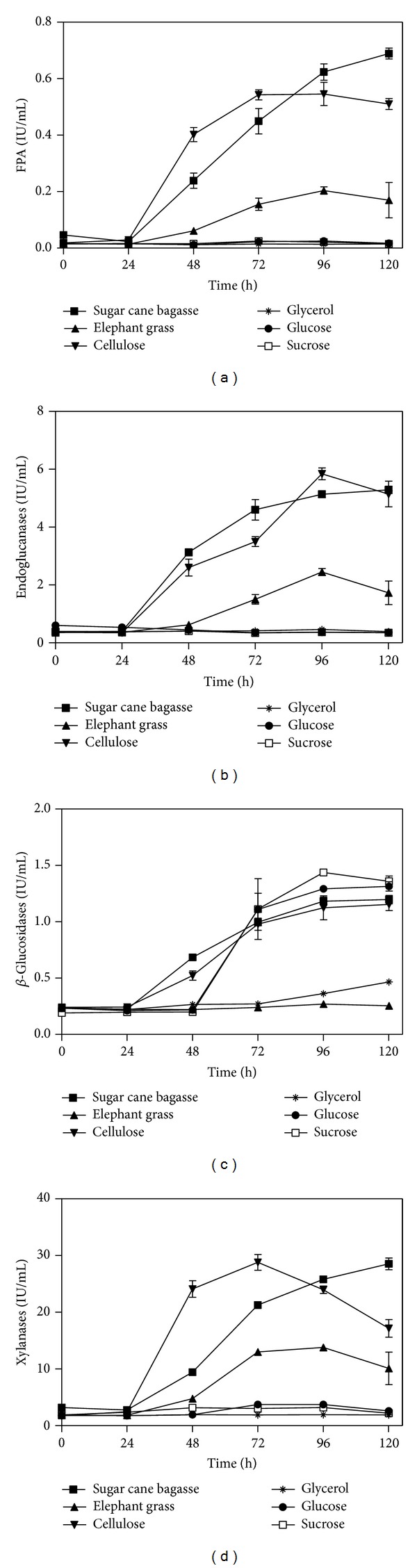
Activities on filter paper (a), endoglucanases (b), *β*-glucosidases (c), and xylanases (d) versus time of* P. echinulatum* S1M29 in submerged cultive, using different carbon sources.

Regarding the enzymatic activity of FPA (Figure 3(a)), it was observed that the culture media using sugar cane bagasse or cellulose were obtained, respectively, the largest enzymatic titles, followed by the medium with elephant grass. It was observed that the increase of enzyme activity from 24 hours of culture, reaching a peak at 120 hours into the medium with sugar cane bagasse (0.69 ± 0.03 IU/mL) and 96 hours for the medium with cellulose (0.59 ± 0.005 IU/mL) and elephant grass (0.20 ± 0.02 IU/mL). The values of FPA with cellulose were lower than those observed by [12], but the latter was obtained by adding sucrose. Delabona et al. (2012) [25] reported obtaining higher activities of FPA (0.78 IU/mL) using bagasse from steam explosion sugar cane. This study used a strain of* Trichoderma harzianum* P49P11 and carbon sources evaluated were sugar cane bagasse, sucrose, glycerol, lactose, and fructooligosaccharides. Juhász et al. (2005) [26] also tested different carbon sources on production of cellulases and xylanases with* T. reesei* Rut-C30. The maximum FPA activity (1.2 IU/mL) was obtained employing cellulose Solka Floc in 168 hours of cultive, indicating that data obtained for* P. echinulatum* have potential when compared to* T. reesei*.

The remaining culture media, employing glucose, sucrose, or glycerol, showed enzymatic activity on filter paper reduced below 0.025 IU/mL, suggesting that these carbon sources do not exhibit inducing effect for producing FPA. These data are according to Ilmén et al. (1997) [27] that observed that, in a study of glucose, fructose, and glycerol as carbon source, production levels obtained insignificant cellulases. Glucose acts as a repressor of cellulase synthesis at the level of transcription in* T. reesei* QM9414. After 45 hours, the lag period of glucose, mRNA levels were detected by northern blot analysis. The inability to provide for expression of cellulases could be explained either by the absence of an inducer that is consumed quickly by the accumulation of glucose.

#### 3.2.2. Endoglucanases Activity

Regarding the activities of endoglucanases (Figure 3(b)) in media containing cellulose and sugar cane bagasse showed the highest enzymatic titles, followed by the medium with elephant grass. It was observed that in the medium with cellulose and elephant grass maximal activities were obtained of 5.84 ± 0.35 IU/mL and 2.45 ± 0.20 IU/mL, respectively, after 96 hours of culture. Medium formulated with sugar cane bagasse yielded maximum activity of 5.29 ± 0.22 IU/mL in 120 hours of cultive. In contrast, the medium formulated with glucose, sucrose, or glycerol showed reduced activity of endoglucanases in comparison with other culture media employed. It is suggested again that glucose, sucrose, and glycerol are not good carbon sources for inducing endoglucanases in* P. echinulatum* S1M29.

These data are consistent with data obtained by Kalra et al. (1984) [28] which suggests that the expression of cellulases does not occur on carbon sources which promote rapid growth as glucose and glycerol but would be caused to more complex sources or disaccharides such as cellulose, cellobiose, and lactose.

#### 3.2.3. *β*-Glucosidases Activity

The results for *β*-glucosidases (Figure 3(c)) showed a different behavior when compared to the other enzymatic activities discussed above. In medium were sucrose (1.43 ± 0.032 IU/mL), glucose (1.29 ± 0.035 IU/mL), sugar cane bagasse (1.19 ± 0.065 IU/mL), or cellulose (1.15 ± 0.09 IU/mL) were used as carbon sources were obtained, respectively, the largest securities enzyme in 120 hours of cultive, with the exception of the medium formulated with sucrose, which reached a peak of activity of *β*-glucosidase in 96 hours of culture.

In culture media with glycerol or elephant grass the lowest activity of *β*-glucosidase was obtained. In the culture medium with glycerol maximal activity was obtained from 0.46 ± 0.035 IU/mL in 120 hours of cultive, while, in medium formulated with elephant grass as a carbon source, there was obtained maximum activity around 0.27 ± 0.018 IU/mL in 96 hours of cultive.

Importantly, the activities of *β*-glucosidase were higher at the end of the process for all media types. This is possibly due to cell lysis that occurs at the end of the enzymatic production process [29].

The low activities of *β*-glucosidases in medium glycerol and elephant grass can be associated at the pH. In the other carbon sources decreases of pH were observed and for the medium with glycerol and elephant grass the pH increased (Figure 1). Reis et al. [30] observed that the highest activities of *β*-glucosidases were found at more acidic pH values (between 5.0 and 6.0). Furthermore the medium with glycerol showed low biomass production and these enzymes occur due cell lysis [29].

#### 3.2.4. Xylanases Activity

As for xylanase activity (Figure 3(d)) in medium formulated with cellulose, sugar cane bagasse and elephant grass again obtained the highest enzymatic titles, respectively. The cultive with cellulose reached maximum activity of 28.8 ± 2.40 IU/mL after 72 hours of culture, followed by cultive with elephant grass, which obtained maximum activity of xylanase in 96 hours of culture, corresponding to 13.8 ± 0.10 IU/mL. The peak of xylanase activity (28.5 ± 1.77 IU/mL) for cultive with sugar cane bagasse was at 120 hours of culture.

The cultures formulated with glucose, sucrose, or glycerol showed enzymatic activity reduced below 4.0 IU/mL, suggesting that these carbon sources show no inducing effect for production of xylanases. The reduced activity observed in these three xylanases culture media may have been due to the absence of substrate (xylan); since that the medium with sugar cane bagasse and elephant grass showed higher activities of xylanases, precisely because these possess hemicellulose in its composition, and the xylan hemicelluloses are more abundant.

The high xylanase activities in medium containing cellulose are probably due to the fact that the cellulase regulator ACEII also influences xylanase regulation. The presence of cellulose may induce not only cellulase production but also the production of xylanase [31].

It is known that in fungi, mainly in* filamentosus* fungi, the lignocellulolytic enzyme production is mightily controlled at the transcriptional level by the competitive action of transcriptional activators and repressors [27].

In the present work, although reduced, xylanase activities in medium formulated with glucose, sucrose, or glycerol were detected. Similar data were reported by Seyis and Aksoz (2005) [32] to the fungus* T. viride* formulated in a medium containing glucose, indicating a possible constitutive production of xylanase. Other studies associated with constitutive xylanase activity in* Trichoderma *sp. [33], showed that basal levels of activity correspond xylanolytic activity of xylanase* xyn2*, whose transcript was not affected by the presence of glucose culture medium, but it would expression levels increased in the presence of xylan, xylobiose and sophorose.

### 3.3. SDS-PAGE of Enzyme Broths Produced by* Penicillium echinulatum* S1M29 in Submerged Cultive

In the analysis of the gels was observed the presence of multiple bands in the samples of enzyme broths produced from different carbon sources used in the culture media.

**Figure 4:**
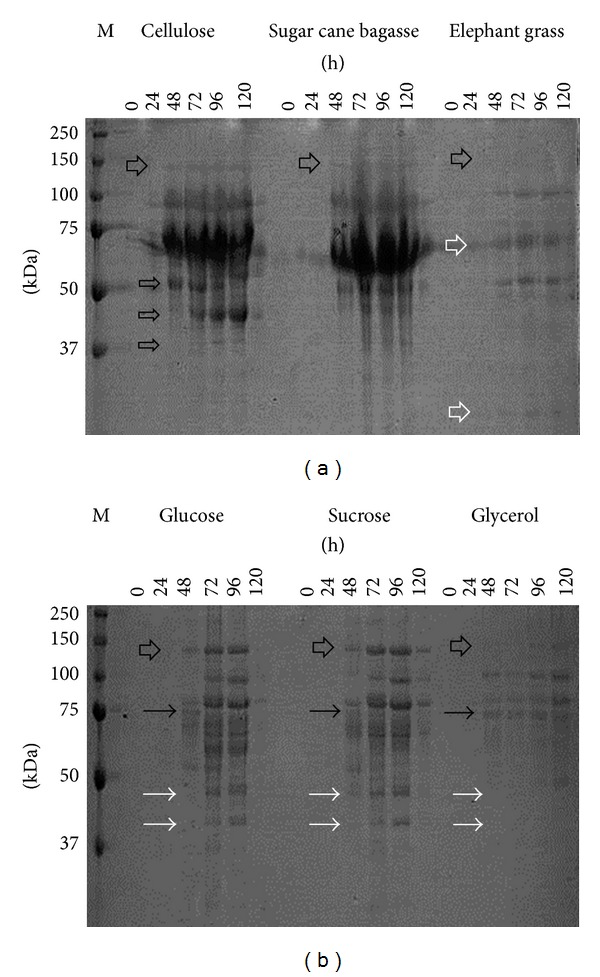
Profile of total proteins separated by gel electrophoresis polyacrylamide (SDS-PAGE) extracted and precipitated from the enzyme broth of* P. echinulatum* S1M29 in submerged culture using different carbon sources. The numbers on the vertical represent the molecular mass protein in kDa (M). The numbers on the horizontally represent the cultive times.

The gel analysis of total proteins (Figure 4) obtained from the enzyme broth of* P. echinulatum *grown in medium with cellulose revealed the presence of numerous bands, between 37 and 150 kDa. Most of these bands were more apparent after 48 hours of cultive, data which corroborate the enzymatic activity. It is important to note that cellulose was the inducer that allowed the detection of early bands. Already in 24 hours some bands have been observed, while other cultive bands were observed only in 48 hours or 72 hours in the case of glucose. Also the presence of bands of 39 and 45 kDa increased the intensity according to the process time and another band (55 kDa) which reduced intensity.

The gel of total proteins obtained from sugar cane bagasse showed a similar standard of bands compared to total protein gel obtained from the cellulose enzyme broth. The bands were more intense between 37 and 150 kDa, and most of these were more evident after 48 hours of cultive. As observed for cellulose in the presence of sugar cane bagasse the presence of diffuse bands was observed, indicating production of glycosylated enzyme.

Few bands were visualized from the medium formulated with elephant grass, and these were most evident after 72 hours of culture, data that corroborate the enzymatic activities. The bands with molecular weight showed increased intensity between 50 and 100 kDa.

The band standards obtained from the broth with glucose and sucrose were similar, except that for sucrose the presence of bands after 48 hours was observed, while for glucose bands were observed only after 72 hours. The protein bands showed less degree of glycosylation when compared to cellulose medium, since the diffuse band is typical of glycosylated proteins in SDS-PAGE [34].

The gel containing the samples from the medium formulated with glucose showed bands between 37 and 150 kDa, and these began to appear after 48 hours but were more evident between 72 and 96 hours of culture; data are consistent with the enzyme activity.

As for the gel formulated with sucrose or glycerol means, the bands showed a molecular mass between 37 and 150 kDa for sucrose and from 50 to 150 kDa glycerol, appearing after 48 hours and intensifying from 72 hours to 120 hours of cultive, data that corroborate the enzymatic activities.

The gel formulated with glucose, sucrose, or glycerol showed sharper bands than the others, suggesting that the presence of these carbon sources secreted proteins is glycosylated less than the media formulated with other carbon sources tested, which showed diffuse bands.

### 3.4. Effect of Carbon Sources over* Penicillium echinulatum* Morphology

The observations in optical microscopy and scanning electron microscopy of* P. echinulatum* S1M29 grown in medium with various carbon sources revealed distinct forms of mycelium, ranging from freely dispersed mycelia to pellets aggregates, suggesting that the composition of the medium with various carbon sources influences the morphogenesis of the fungus.

**Figure 5:**
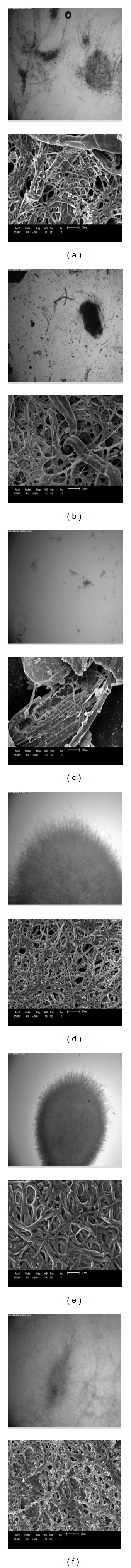
Optical microscopy images (upper panels) and scanning electron microscopy images (lower panels) of* P. echinulatum* S1M29 in 48 hours of submerged cultive, using cellulose (a), sugar cane bagasse (b), elephant grass (c), glucose (d), sucrose (e), and glycerol (f) as carbon sources.

**Figure 6:**
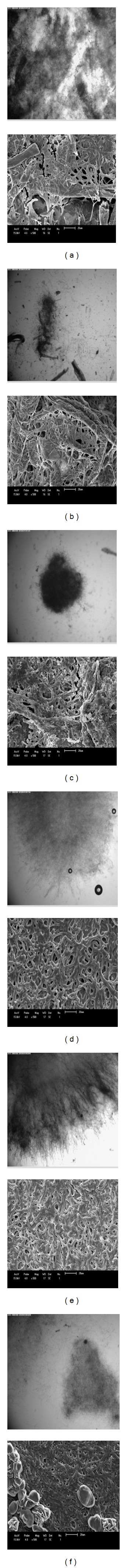
Optical microscopy images (upper panels) and scanning electron microscopy images (lower panels) of* P. echinulatum* S1M29 in 72 hours of submerged cultive, using cellulose (a), sugar cane bagasse (b), elephant grass (c), glucose (d), sucrose (e), and glycerol (f) as carbon sources.

The medium formulated with cellulose (Figures 5(a) and 6(a)) presents a dispersed morphology, ranging from freely dispersed and aggregated (or mycelial clumps) as there are many regions with overlapping hyphae. Although it is difficult to identify the main hyphae, there is great branch thereof, which assume a morphology of hyphae elongated involving large part of the substrate surface, thereby increasing the interaction between the mold and the substrate. Ahamed and Vermette (2009) [35] found similar morphology in liquid media formulated with cellulose using* T. reesei* Rut-C30, whereas this morphology characterized by a highly branched hyphae elongated and has been associated with good productivity enzyme depending on the potential increase of the interaction between large amounts of branching hyphae and the substrate of the medium.

It is suggested, therefore, that a more dispersed morphology is conducive to enzyme secretion, as can be seen in the medium formulated with cellulose.

The morphology of the medium formulated with sugar cane bagasse (Figures 5(b) and  6(b)) is very similar to the medium with cellulose, characterized by being a dispersed morphology with large branching hyphae. Long and branched hyphae increase the surface area of the fungus, possibly increasing the interaction with the substrate and thus the productivity of the enzyme [36].

The images of scanning electron microscopy of the medium prepared with elephant grass are fully compatible with the images obtained by optical microscopy and the results of enzymatic activities. As can be seen in Figure 5(c), up to 48 hours of culture, branching hyphae is less when compared to cellulose and sugar cane bagasse, prevailing waste of elephant grass present in the culture medium.

However, at 72 hours of cultive (Figure 6(c)), hyphae covering large surface area of the substrate, with large overlapping hyphae, assumes a pellet morphology form and it is difficult to visualize spaces between the entangled hyphae.

The low enzymatic activities obtained in medium with cultivated elephant grass can also be related to compounds that interfere with the fungal enzyme secretion, from the decomposition of biomass itself elephant grass. Recently it was investigated the effect of phenolic monomers on the growth of* Acidothermus cellulolyticus*, and it was reported that some of the phenolic compounds present in elephant grass were toxic to* A. cellulolyticus*, by inhibiting phenolic [37].

Microscopy of media formulated with glucose, sucrose, or glycerol showed a very similar morphological definition to each other. The microscopic analysis revealed that after 48 hours of cultive the fungus appeared thick and branched entangled with large overlapping hyphae; it is impossible to identify a primary hyphae of all others that were intertwined and/or interconnected.

In accordance with the pH data, the media formulated with sucrose and glucose showed a significant reduction in pH after 24 hours of culture, at which both media showed an increase in mycelial mass. It is suggested, once again, that the drop in pH is indicative of an increase in cellular metabolism of the fungus, at which time it begins to consume the substrate present in the medium and enhance their growth.

However, at 72 hours of cultive, hyphae were also denser and branched, which agrees with data obtained from the growth in which the amount of mycelial mass in 72 hours was over 48 hours, characterizing their morphology in form of pellets, which can already be seen in optical microscopy. It is suggested, once again, that the morphology in pellets form for the fungus* P. echinulatum* S1M29 inhibits enzyme secretion, once the media formulated with sucrose or glucose alone led to high production of *β*-glucosidases.

Analyzing Figures 5(d) and 5(e), thicker hyphae can be observed when compared to other cultive media. It is possible to identify new tips hyphae only in the medium formulated with glucose, although, in this situation, this parameter is not indicative of higher productivity enzymatic activities because media formulated with glucose, sucrose, or glycerol were reduced, except for the activities of *β*-glucosidases media containing glucose or sucrose, which were more expressive. The reduced activities can be explained by catabolite repression [38, 39].

Different from that observed in the present study, Nitta et al. (2012) [40] obtained thicker hyphae in cultures containing Avicel than in glucose cultures using* T. reesei*.

For* P. echinulatum* in the presence of cellulose (Figure 5(a)) were observed hyphae thinner than hyphae in the presence of sucrose (Figure 5(e)) or cane sugar bagasse, showing that there are also physiological and morphological differences relative to* T. reesei* QM9414.

A study of Cho et al. (2001) [41] on the influence of sucrose concentration on the morphology of* Paecilomyces japonica* showed that the pellet size increased with increasing sucrose concentration. However, with an initial substrate concentration of 80 g/L, there was observed the formation of pellets and the entire population of fungi was characterized by filamentous growth.

In a review study conducted by El-Enshasy (2007) [42], it was reported that the morphology in the form of pellet is not necessarily detrimental to any fungus. This ratio generally varies between different strains of fungi and even between different products from the same strain. For example, the growth in the pellet form is preferred for producing the precursor pravastatin by* Penicillium citrinum*, citric acid by* Aspergillus niger*, and* Rhizopus chinensis* antibiotics as dispersed growth is preferable for penicillin production by* P. chrysogenum*, fumaric acid by* Rhizopus arrhizus*, and enzymes by* A. niger*.

Moreover, production of heterologous proteins in* A. niger* was greater in pelletized form. This form of growth inhibits the production of extracellular protease and thereby indirectly increases the production of heterologous protein. Besides the growth morphology, spatial distribution, the arrangement of the cells within the pellet, the structure of the surface, and the cell density inside the pellet may also influence product formation. The differentiation during the formation of mycelial pellets also has marked effects on enzyme production. Production of polygalacturonase by* A. niger* is well correlated with the mycelial morphology; the more compact the pellet is, the more enzyme is synthesized. A high yield of antibiotic production in* R. chinensis* is related to the formation of a pellet less compact, softer, and looser.

Therefore, there is no general theory for this relationship. Some products are highly induced when growth is in filamentous form; others are expressed at high titers when growth is pellet.

## 4. Conclusions

The fungus* P. echinulatum* showed differential growth in different carbon sources employed, demonstrating that the composition of the culture medium interferes in the synthesis of proteins which are secreted and in the morphology of mycelia. In the case of cellulose, an insoluble substrate, the area available for the hypha nutrient absorption and secretion is significantly reduced when forming pellet and must compromise the secretion of cellulases and xylanases. The data obtained in this study contribute to the understanding of fungal physiology and contribute to formulating means and conditions of fermentation processes for obtaining broths enzyme with potential application in the technology of second generation ethanol.
